# Dr. Ducros’ Experimental Researches on the Function of the Skin in Man and Animals

**Published:** 1842-05-07

**Authors:** 


*Experimental Researches on the Function of the Skin in man and animals.* By Dr. Ducros.—In a very curious experimental paper, Dr. Ducros shows that a coating of gum-lac put on the skins of animals causes them to die in a longer or shorter time, by producing convulsive movements similar to epilepsy. When the animals, coated with gum-lac, were subjected to electricity, they died in a much shorter time. He next tried the effect of metallic coverings, as he entertained the notion that, because they had opposite electrical properties, the animals coated with them would die with symptoms of an opposite nature. He therefore cut off the hair from some animals and covered them with thin plates of tin, (tin-foil) and found that they perished with symptoms of debility, the reverse of what he had noticed when the coating consisted of a resinous substance. When the tin was covered with a coating of gum-lac, the animals perished still more rapidly. He then placed under the influence of electricity some of the animals covered with plates of tin, and found that so long as they remained connected with the electrical current, their vigour appeared to be restored, but that, whenever it was arrested, they appeared ready to perish.

The object of these experiments was to ascertain what would be the likely effect of such coverings in certain diseased states of the human frame, and especially in nervous or neuralgic affections, and in rheumatism. He reasoned, that, if metallic coverings deprived animals of life by producing rapid sinking of the vital powers, the same metallic plates applied to the human body would cure or remove those diseases which seemed to depend on an excess of organic life. On putting his plan to the test of practice, he was so fortunate as to find that it removed some nervous, and a few acute and chronic rheumatic affections.

This plan of treatment was of no avail in any case where the disease was dependant on or connected with organic lesions, or attended with fever, or swelling of the part, or with general weakness; on the contrary, in all these cases the metallic plates augmented the disorder.—*Ed. Med. and Surg. Jour.*, from *Comptes Rend. des Sean. de l'Acad. des Sci.*, 20th Sept., 1841.

